# A differential autophagy-dependent response to DNA double-strand breaks in bone marrow mesenchymal stem cells from sporadic ALS patients

**DOI:** 10.1242/dmm.027938

**Published:** 2017-05-01

**Authors:** Shane Wald-Altman, Edward Pichinuk, Or Kakhlon, Miguel Weil

**Affiliations:** 1 Laboratory for Neurodegenerative Diseases and Personalized Medicine, Department of Cell Research and Immunology, The George S. Wise Faculty for Life Sciences, Sagol School of Neurosciences, Tel Aviv University, Ramat Aviv, Tel Aviv 69978, Israel; 2 Department of Neurology, Hadassah-Hebrew University Medical Center, Ein Kerem, Jerusalem 91120, Israel

**Keywords:** ALS, DNA damage response, Autophagy, Human mesenchymal stem cell

## Abstract

Amyotrophic lateral sclerosis (ALS) is an incurable motor neurodegenerative disease caused by a diversity of genetic and environmental factors that leads to neuromuscular degeneration and has pathophysiological implications in non-neural systems. Our previous work showed abnormal levels of mRNA expression for biomarker genes in non-neuronal cell samples from ALS patients. The same genes proved to be differentially expressed in the brain, spinal cord and muscle of the SOD1^G93A^ ALS mouse model. These observations support the idea that there is a pathophysiological relevance for the ALS biomarkers discovered in human mesenchymal stem cells (hMSCs) isolated from bone marrow samples of ALS patients (ALS-hMSCs). Here, we demonstrate that ALS-hMSCs are also a useful patient-based model to study intrinsic cell molecular mechanisms of the disease. We investigated the ALS-hMSC response to oxidative DNA damage exerted by neocarzinostatin (NCS)-induced DNA double-strand breaks (DSBs). We found that the ALS-hMSCs responded to this stress differently from cells taken from healthy controls (HC-hMSCs). Interestingly, we found that ALS-hMSC death in response to induction of DSBs was dependent on autophagy, which was initialized by an increase of phosphorylated (p)AMPK, and blocked by the class III phosphoinositide 3-kinase (PI3K) and autophagy inhibitor 3-methyladenine (3MeA). ALS-hMSC death in response to DSBs was not apoptotic as it was caspase independent. This unique ALS-hMSC-specific response to DNA damage emphasizes the possibility that an intrinsic abnormal regulatory mechanism controlling autophagy initiation exists in ALS-patient-derived hMSCs. This mechanism may also be relevant to the most-affected tissues in ALS. Hence, our approach might open avenues for new personalized therapies for ALS.

## Abstract (precis)

**Summary:** A novel endogenous disease mechanism in cells from ALS patients after NCS-induced DNA damage.

## INTRODUCTION

Amyotrophic lateral sclerosis (ALS) is a neurodegenerative disease characterized by progressive muscular paralysis reflecting degeneration of motor neurons (MNs) in the primary motor cortex, brainstem and spinal cord. Denervation results in muscle weakness, atrophy and spasticity that lead to a failure in initiating and controlling voluntary movements (Mizutani et al., 1992). About 90% of ALS cases are sporadic (sALS) and absent of any apparent genetic linkage (Pasinelli and Brown, 2006). Between 5 and 10% of ALS cases are reported to be familial ALS (fALS) (Byrne et al., 2011; Mulder et al., 1986; Gros-Louis et al., 2006). Mutations in at least 126 genes (ALSoD Online genetics Database, 2016; http://alsod.iop.kcl.ac.uk) are now recognized to be linked with most fALS cases, with some mutations even linked with sALS. This abundance of genes indicates multifactorial causes for the disease (Eisen, 1995). Given the diversity of factors causing the disease, it is reasonable to think that ALS is a product of multiple abnormal molecular pathways and intracellular mechanisms that lead to not only to MN degeneration but in parallel also affect other cell types and systems, probably exacerbating ALS pathology. Supporting this view is the finding that astrocytes derived from fALS and sALS patients were toxic to MNs (Haidet-Phillips et al., 2011), together with evidence showing that thymic involution leading to immunodeficiency contributes to ALS pathology (Seksenyan et al., 2010). Since degenerating MNs cannot be harvested from ALS patients for research, there are great benefits to using non-neural cell types like human mesenchymal stem cells (hMSCs) (Nachmany et al., 2012; Lilo et al., 2013; Kassis et al., 2013), which are readily isolated from tissues by less-invasive procedures and can be harvested in culture in large quantities without the need for re-sampling, for experimental purposes. The importance of such human ALS patient cell sample models for research is that they keep both the genetic background and their intrinsic epigenetic makeup that may have contributed to the development of ALS in the patient. Together with this, recent advances in producing MNs from somatic cells of ALS patients by induced pluripotent stem cells methodologies (Devlin et al., 2015; Dimos et al., 2008) may help to investigate relevant cellular neurodegenerative mechanisms in respect to the genetic makeup of the patient. Our previous work in human mesenchymal stem cells (hMSCs) isolated from the bone marrow of several sALS patients (ALS-hMSCs) identified four ALS biomarkers, CyFIP2, RbBP9, TDP-43 (also known as TARDBP) and SLPI, that showed abnormal mRNA expression levels (Nachmany et al., 2012; Lilo et al., 2013). Moreover, these genes also show differential expression in brain, spinal cord and muscle in the SOD1^G93A^ ALS mouse model supporting, on one hand, the pathophysiological relevance of the ALS biomarkers discovered in non-neuronal samples of sALS patients and, on the other, the potential of ALS-hMSCs to be a useful model to study the intrinsic cell molecular mechanism of the disease. Establishing hMSCs as a relevant ALS model is also very important because of their therapeutic potential for treating neurodegenerative diseases, which warrants their use in mechanistic studies of neurodegenerative diseases. Several lines of evidence indicate that oxidative stress induces DNA damage in ALS. ALS cells were found to sustain higher accumulation of DNA breaks in spinal cord and brain samples from ALS patients (Sau et al., 2007; Kisby et al., 1997; Kikuchi et al., 2002) as well as in neurons of several ALS mouse models (Cai et al., 2005; Suraweera et al., 2007). The biological implications of this unique propensity for DNA damage in ALS cells and tissues remain obscure. To test whether DNA damage has a role in ALS, we investigated the response of the ALS-hMSCs to oxidative DNA damage, adopting the well-established effect of neocarzinostatin (NCS) to induce double-strand breaks (DSBs) to cause stress; this leads to similar biological effects to those induced by ionizing radiation and involves the known DNA repair molecular cascade (Kuo et al., 1984; Banin et al., 1998). Here, we show that ALS-hMSCs from several sALS patients, cultured for 24 h in defined conditions, respond differently to DSB-induced stress compared to hMSCs from healthy controls (HC-hMSCs). Interestingly, we found that the known signaling components of the early DNA damage response are present at a similar location and time between ALS-hMSCs and HC-hMSCs. However, only ALS-hMSCs concomitantly activate autophagy-mediated cell death, which is blocked by the class III PI3K and autophagy inhibitor 3-methyladenine (3MeA). This unique ALS-hMSC-specific response to DNA damage indicates that an intrinsic abnormal regulatory mechanism controlling cytotoxic autophagy initiation exists in sALS-patient-derived hMSCs. This result emphasizes the viability of using ALS-patient-derived non-neuronal cells, like hMSCs, in searching for intrinsic disease-related biomarkers and disease molecular pathways that may also be relevant to the most-affected tissues in ALS. Hence, this approach might open avenues for new personalized therapies for this incurable and devastating disease.

## RESULTS

### DNA damage response elicits cell death in ALS-hMSCs and not in HC-hMSCs

**Fig. 1.**
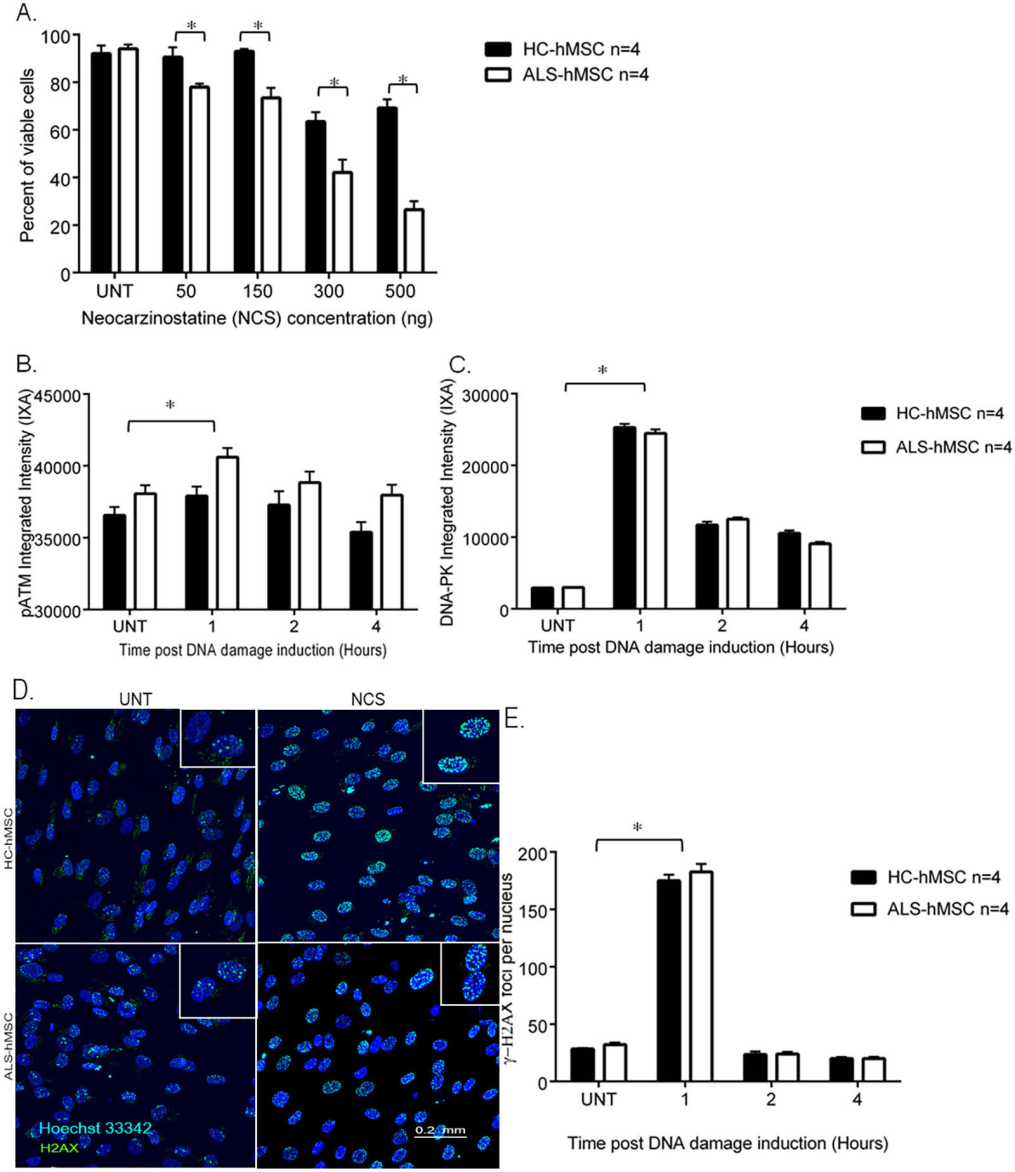
**Characterization of the NCS-induced DNA damage response in terms of cell viability and early induction of DSB sensors in ALS- and HC-hMSC populations.** (A) The cells were treated with 50, 150, 300 and 500 ng/ml NCS for 30 min to induce DNA damage, rinsed and then kept in culture with fresh medium for 24 h. Cells were then fixed and the DNA damage response in terms of cell viability was quantified for the total number of nuclei using a living and dead fluorescence assay by staining the cells in microscopy-grade 96-well plates with a mix of Calcein-AM for live cells, propidium iodide (PI) for dead cells, and Hoechst 33342 to label all nuclei. Multiple images of thousands of cells were taken for image analysis of cell viability using the INCell 2000 image analyzer and software. Shown are mean±s.e.m. of a group of *n*=6 HC-hMSCs and *n*=6 ALS-hMSCs. For each individual, at least three independent experiments, where 10,000 cells were analyzed, were conducted. *P<0.05. (B–E) Cells were treated with 50 ng/ml NCS, and induction of known DSB sensors at 1, 2 and 4 h post NCS treatment was quantified. Immunofluorescence analysis of pATM (B), pDNA-PK (C) and γ-H2AX (E) levels was undertaken by measuring the integrated intensity (IXA) of the signal in the nuclei post NCS treatment in ALS-hMSCs and HC-hMSCs. The mean±s.e.m. of 10,000 cells per individual were analyzed from the images from at least three independent experiments of a group of HC-hMSCs (*n*=4) and ALS-hMSCs (*n*=4). *P<0.05. (D) Representative images of stained nuclei showing γ-H2AX-labeled foci before and 1 h after NCS treatment in ALS-hMSCs and HC-hMSCs. UNT, untreated cells.

**Fig. 2.**
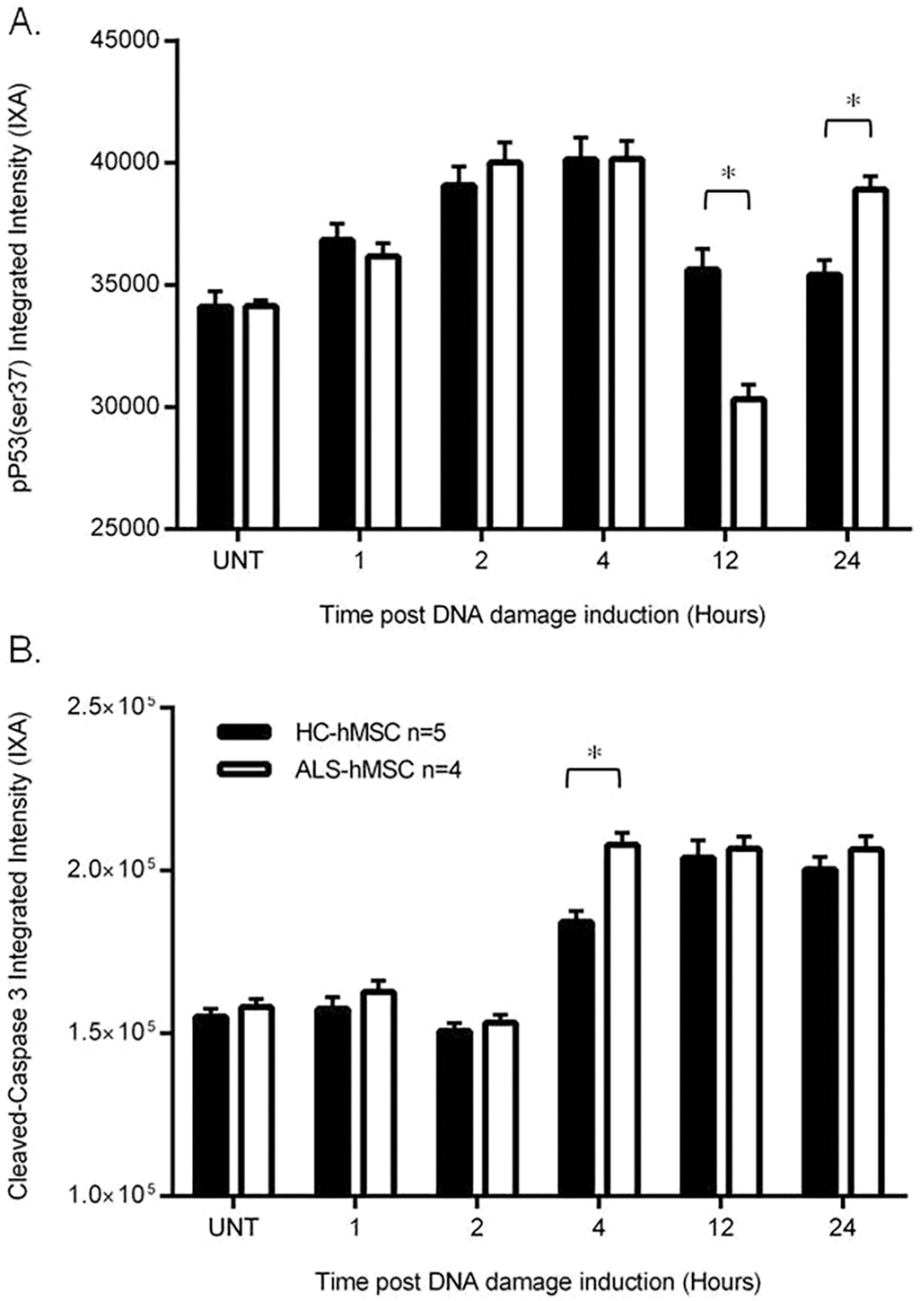
**Levels** **of p-p53Ser37 and** **the apoptosis effector** **cleaved caspase-3 in ALS- and HC-hMSCs subjected to NCS-induced DNA damage.** The cells were treated with 50 ng/ml NCS and incubated for 1, 2, 4, 12 and 24 h then fixed and prepared for immunofluorescence microscopy analysis of p-p53Ser37 and for cleaved caspase-3 expression using specific antibodies. (A) Quantitative results of the nuclear integrated intensity (IXA) the of p-p53Ser37 signal after NCS-induced DNA damage in ALS-hMSCs and HC-hMSCs. (B) Quantitative results of the cytoplasmic cleaved caspase 3 IXA in the same parallel experiments as in A. The mean±s.e.m. of 10,000 cells per individual was analyzed from the images from at least three independent experiments of a group of HC-hMSCs (*n*=5) and ALS-hMSCs (*n*=4). *P<0.05. UNT, untreated cells.

To study the stress response to NCS-induced DNA damage in ALS-hMSCs and HC-hMSCs, we cultured primary hMSCs from various donors in serum-free conditions for 3 days to allow optimal treatment evaluation. As previously shown by us, hMSCs are able to survive and remain in the G0/G1 state for prolonged periods of time (Solmesky et al., 2010). At first, we treated the cells for 24 h with NCS at 50, 150, 300 and 500 ng/ml and evaluated the effect of NCS on cell viability by image-based fluorescence microscopy high-content analysis (HCA), using a mix of vital fluorescent dyes (calcein-AM, propidium iodide and Hoechst 33342) to identify living and dead cells as described previously (Solmesky et al., 2010). Interestingly, in these experiments NCS treatment differently affected the cell viability of ALS-hMSCs (*n*=4) in a significant manner as compared to HC-hMSCs (*n*=4) at every tested NCS concentration and in a concentration-dependent manner (Fig. 1A). To investigate further the mechanism responsible for this difference in cell viability to the DNA damage response in the ALS-hMSCs population, we decided to focus on the effects induced by the lowest dose of NCS (50 ng/ml) in this process. We first investigated the effects of NCS on triggering the sensory stage of the DSB-induced DNA damage response by following the level of the activated phosphorylated (p)ATM and DNA-dependent protein kinase (DNA-PK). These are the major kinases that are sequentially activated in cells during the first 4 h upon sensing DNA DSBs, which in turn activate γ-H2AX (Lee and Paull, 2007; Stiff et al., 2004; Harper and Elledge, 2007). ALS-hMSCs and HC-hMSCs were plated in microscopy-grade 96-well plates, then treated with 50 ng/ml NCS for 1, 2 and 4 h. Cells were fixed with 4% paraformaldehyde (PFA) and immunofluorescence analysis was performed by using specific antibodies to detect the active phosphorylated forms, namely, anti-pATM (Ser1981), anti-pDNA-PK (Ser2056) and anti-γ-H2AX (Ser139) antibodies. We quantified the intracellular fluorescence intensity of these proteins by HCA immunofluorescence with the InCell 2000 Image analyzer. An average of images from 10,000 cells per individual, for both ALS and HC, were analyzed from three independent experiments, for each of the *n*=4 subjects analyzed by the software, with the integrated intensity (IXA; intensity multiplied by the cell area) of pATM or pDNA-PK calculated as shown in Fig. 1B,C, respectively. Representative images showing the difference in the number of γ-H2AX-labeled DSB foci in the nuclei of ALS-hMSCs and HC-hMSCs before and 1 h after NCS treatment are shown in Fig. 1D. Differences in the number of γ-H2AX foci are quantified in Fig. 1E. In both ALS-hMSCs and HC-hMSCs, pATM and pDNA-PK protein expression levels significantly increase 1 h post NCS induction, decreasing back to almost the basal level at 2 to 4 h post NCS induction, as expected (Fig. 1B,C, respectively). In parallel, the number of γ-H2AX foci significantly increased at 1 h post NCS induction in both groups, before decreasing to the basal level by 2 h. Taken together, these results indicate that there is no significant difference in the response that senses DNA damage induced by NCS between HC-hMSCs and ALS-hMSCs, which implies that the observed decrease in cell viability of ALS-hMSCs at 24 h post DNA damage induction is not due to failure in the early sensing-stage machinery, but rather that there is a later signal transduction event that mediates the cell death pathway in the diseased cells and not in HC cells. To test this hypothesis, we performed similar experiments using HCA to analyze the involvement of the key effector p53 (also known as TP53) in the DNA damage response induced by NCS in the ALS and HC cells. We followed p53 activation (through monitoring its phosphorylation on Ser37) induced by either pATM or pDNA-PK for 24 h, which, in turn, is known to activate downstream targets for inducing cell cycle arrest, DNA repair or cell death pathways (Siliciano et al., 1997; Banin et al., 1998; Canman et al., 1998; Khanna et al., 1998; Khosravi et al., 1999; Lambert et al., 1998; Ashcroft et al., 1999). In these experiments, the cells were stained with an antibody against p53 phosphorylated on Ser37 (p-p53Ser37) and in parallel with anti-cleaved caspase 3 to detect, simultaneously with the p53 signal, apoptosis in a cell-by-cell analysis. Fig. 2 shows the results from these experiments. In Fig. 2A a significant increase in the p53Ser37 nuclear integrated intensity (IXA) levels is detected for the first 4 h post DNA damage induction in both HC-hMSCs and in ALS-hMSCs. However, at 12 h post DNA damage induction, p-p53Ser37 levels significantly decrease in ALS-hMSCs as compared to those in HC-hMSCs and in untreated ALS-hMSCs controls. Interestingly, at 24 h post DNA damage induction, we reveal a significant increase in p-p53Ser37 signal in the ALS-hMSCs as compared to that in the HC-hMSCs. In these experiments, we also found that there was a similar level of cleaved-caspase-3 protein in both ALS-hMSCs and HC-hMSCs except for a significant increase at 4 h post DNA damage induction in the ALS patient cells (Fig. 2B). Interestingly, these results suggest that the expression pattern differences for the effector molecule p-p53Ser37, together with those of the apoptosis effector cleaved-caspase-3, between the ALS and HC-hMSCs populations after induction of DNA damage cannot explain the specific and significant decrease in cell viability observed in the ALS-hMSCs population at 24 h post NCS treatment (see Fig. 1A).

### A dysregulated autophagy response accounts for the ALS-hMSC-specific sensitivity to DNA damage

**Fig. 3.**
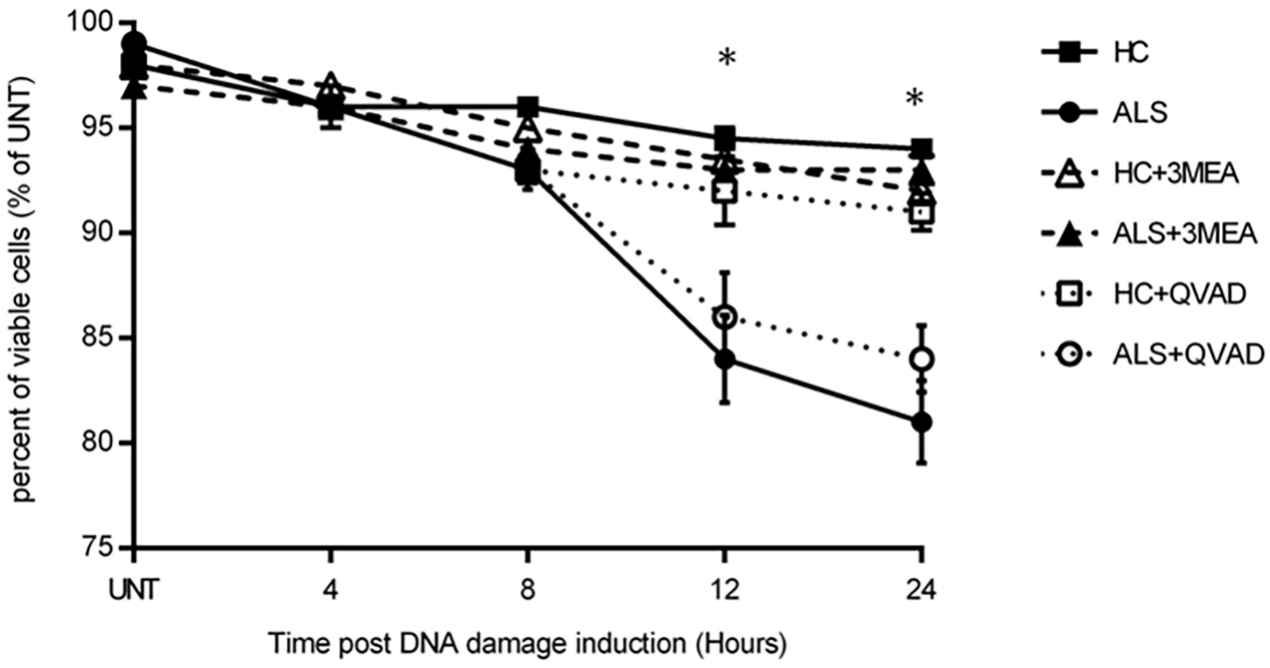
**Effect of the NCS-induced DNA damage response on cell viability in ALS-hMSCs in the presence or absence of the autophagy and caspase inhibitors 3MeA and Q-VAD-OPH.** The cells were treated with 50 ng/ml NCS and were incubated for 1, 2, 4, 12 and 24 h post DNA damage induction in the presence or absence of the apoptosis inhibitor Q-VAD-OPH at 20 μM or of the autophagy inhibitor 3MeA at 10 mM. Cell viability was measured using a living and dead fluorescence assay by staining the cells in microscopy-grade 96-well plates at indicated time points with a mix of Calcein-AM for live cells, propidium iodide (PI) for dead cells, and Hoechst 33342 to label all nuclei. Multiple images of thousands of cells were taken for image analysis of cell viability using the INCell 2000 image analyzer and software. The percentage of viable cells was calculated from total nuclei and is presented relative to the value from the respective untreated control (UNT). Cells were incubated with 3MeA or Q-VAD-OPH for 1 day or 30 min prior to NCS induction, respectively. The mean±s.e.m. of 10,000 cells per individual was analyzed from images from at least three independent experiments of a group of HC-hMSCs (*n*=6) and ALS-hMSCs (*n*=6). *P<0.05 between HC and ALS and between ALS and ALS+3MeA treatments were observed after 12 h of DNA damage induction.

To investigate further the possible cell death mechanisms involved in triggering cell death specifically in the ALS cell population, we performed cell viability experiments in a way similar to that described above (Fig. 1A). Here, ALS-hMSCs (*n*=6) and HC-hMSCs (*n*=6) were exposed to 50 ng/ml NCS for 4, 8, 12 and 24 h in the presence or absence of either a specific pan-caspase inhibitor Q-VAD-OPH at 20 µM or the autophagy inhibitor 3-methyladenine (3-MeA) at 10 mM. The results from these experiments are shown in Fig. 3 as the proportion of live cells in the cell population. ALS-hMSCs show a significant decrease in cell viability from 12 h post NCS induction as compared to that of HC-hMSCs. Moreover, this decrease in cell viability is unaffected by the caspase inhibitor Q-VAD-OPH but is remarkably blocked by the autophagy inhibitor 3-MeA. These results suggest that the disease-related cell death response to NCS-DNA damage in ALS-hMSCs is mediated through autophagy and not by a caspase-dependent pathway. Moreover, these results suggest that there is an abnormal regulatory mechanism in the autophagy pathway in ALS-hMSCs in response to DNA damage.

**Fig. 4.**
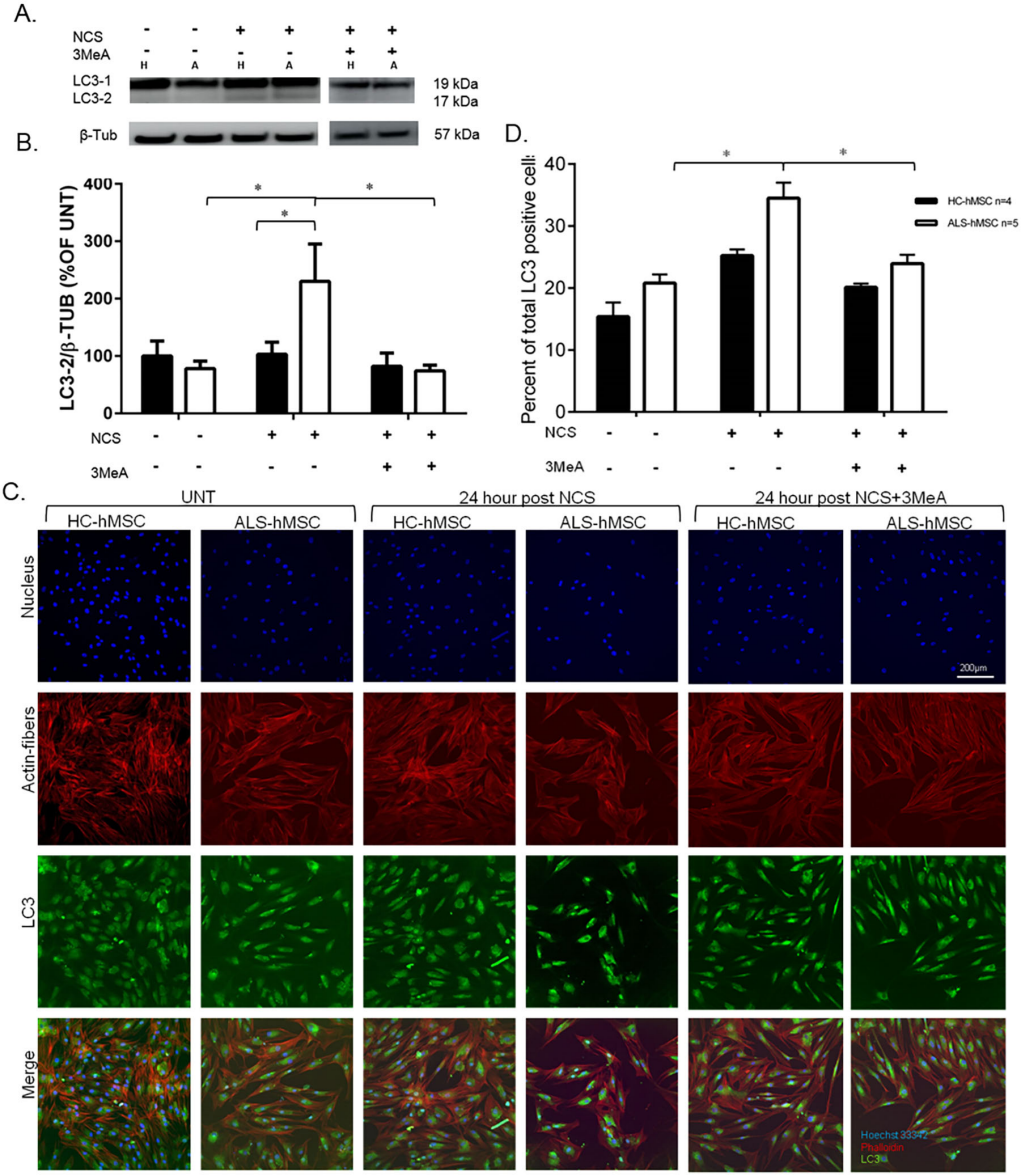
**Effect of the NCS-induced DNA damage response on the LC3 autophagy marker in ALS-hMSCs in the presence or absence of 3MeA.** Western blot analysis or quantitative immunofluorescence analysis of LC3 levels was performed in ALS-hMSCs and HC-hMSCs that were treated with 50 ng/ml NCS or were untreated (UNT) in the presence or absence of 10 mM 3MeA for 24 h. (A) Representative WB showing, in extracts of HC-hMSCs (labeled H) and ALS-hMSCs (labeled A), LC3-I (LC3-1, 19 kDa), LC3-II (LC3-2, 17 kDa) and β-tubulin (β-Tub), as a loading control. (B) Quantitative (mean±s.e.m.) densitometry results of three independent experiments on ALS hMSCs (white bars, *n*=6) and HC-hMSCs (black bars, *n*=6), showing the LC3-2:β-TUB ratio, which is indicative of autophagy. *P<0.05. (C) Representative images of HC-hMSCs and ALS-hMSCs that were untreated or treated with NCS with and without 3MeA after 24 h in culture. Cells were triple-stained with Hoechst 33324 to label the nuclei (blue), phalloidin to label actin fibers (red) and with anti-LC3 antibodies to label LC3 within cells (green). Respective merged color images are shown in the lower panel. (D) Quantitative (mean±s.e.m.) image analysis of the percentage of LC3-positive cells in the cell population measured as the LC3 integrated intensity levels per cell, showing that there is higher signal level as compared to the untreated control for the same individual in each separate group (HC or ALS). An average of 10,000 cells was analyzed per individual, ALS hMSCs (*n*=5), HC-hMSCs (*n*=4). *P<0.05 after 24 h of DNA damage induction between ALS-hMSCs NCS-treated cells and both ALS-hMSCs untreated controls and ALS-hMSCs NCS+3MeA treatments.

**Fig. 5.**
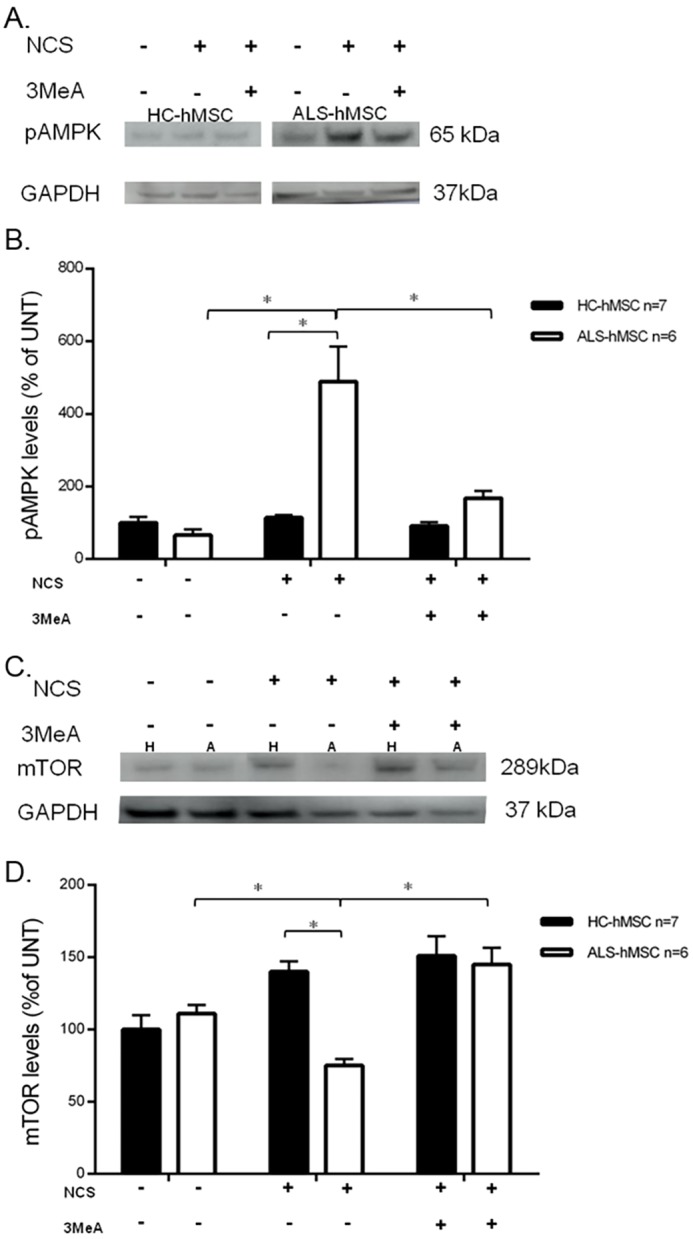
**Effect of NCS-induced DNA damage on autophagy-dependent expression of pAMPK and mTOR protein in ALS-hMSCs.** WB analysis for pAMPK and mTOR was performed in protein extracts from ALS-hMSCs (*n*=6) and HC-hMSCs (*n*=7) treated with 50 ng/ml NCS or untreated in the presence or absence of 10 mM 3MeA for 24 h. (A) Representative WB of pAMPK levels in cells from single HC and ALS individuals. (B) Quantitative densitometry results (mean±s.e.m.) of three independent experiments on ALS-hMSCs and HC-hMSCs, showing the changes in pAMPK levels. (C) Representative WB of mTOR levels in cells from one HC (labeled H) and ALS individual (labeled A). (D) Quantitative densitometry results (mean±s.e.m.) of three independent experiments on ALS-hMSCs and HC-hMSCs, showing the changes in mTOR levels. Levels of pAMPK and mTOR protein expression was normalized to GAPDH and to the level in untreated HC-hMSCs (set as a 100% reference). *P<0.05.

To investigate the possibility that the autophagy pathway is abnormally regulated in ALS-hMSCs in response to DNA damage, we tested differences in key molecular components of the autophagy pathway in ALS-hMSCs compared with that in HC-hMSCs at 24 h post 50 ng/ml NCS treatment with or without treatment with 10 mM 3MeA (Fig. 4). First, we tested the level of the autophagy marker LC3-II (the lipid-modified form of LC3, which comprises the three family members MAP1LC3A, MAP1LC3B and MAP1LC3C), by following previously published autophagy guidelines (Klionsky et al., 2016), by western blot (WB) analysis as shown in Fig. 4A. A significant increase in the amount of total LC3-II normalized to β-tubulin was found in ALS-hMSCs as compared to in HC-hMSCs at 24 h post NCS treatment, which was blocked by the presence of 3MeA (Fig. 4A,B). Interestingly, in these experiments an identical result was obtained for the LC3-II to LC3-I ratio (LC3-I is the cytosolic form of LC3) supporting the idea that there is an increase in intracellular autophagy activity in ALS-hMSCs due to the NCS-induced DNA damage stress (data not shown). In parallel, we tested autophagy changes in cells by using image-based HCA to directly measure LC3 immunofluorescence intensity levels from all cells, as shown in Fig. 4C,D. Representative images of LC3 immunofluorescence staining in the cells show qualitatively the apparent intensity differences in LC3 levels between ALS and HC-hMSCs in response to 24 h DNA damage induction (Fig. 4C). The percentage of cells expressing LC3 increases significantly after 24 h of NCS treatment in both ALS and HC cells as compared to in untreated controls. However, in ALS-hMSCs, the relative response to NCS in terms of autophagy induction was higher than in HC-hMSCs. In both cases, autophagy induction was blocked in the presence of 3MeA (Fig. 4D). Taken together, these results strongly support the view that autophagy is differentially induced in ALS-hMSCs after DNA damage induction by NCS, suggesting that autophagy is regulated differently in the hMSCs isolated from the bone marrow of several sALS patients. 3MeA inhibits autophagy by blocking autophagosome formation via the inhibition of class III PI3K (Wu et al., 2013), which in turn affects mTOR activity, directly regulating the autophagy process. The rescue effects of 3MeA on autophagy [LC3-II-to-β-tubulin ratio and LC3-positive cells (Fig. 4)] and especially on cell death (Fig. 3) may indicate an ALS-hMSC-specific aberrant regulation of autophagy causing it to be cytotoxic, rather than cytoproective as it is in HC-hMSCs. The aberrant regulation of the autophagy pathway in the ALS-hMSCs is therefore upstream of mTOR and LC3, at the level of autophagosome formation, which is directly inhibited by 3MeA (Axe et al., 2008). We next hypothesized that the DNA damage response abnormally induces the autophagy pathway in ALS-hMSCs through AMPK phosphorylation signaling, which inhibits the negative regulator of autophagy mTOR and activates the autophagosome (omegasome) formation factor ULK1 (Kim et al., 2011). To test our hypothesis, we performed WB analysis on protein extracts from each ALS-hMSC (*n*=6) and HC-hMSC (*n*=7) to determine the levels of the phosphorylated (p)AMPK (α2 subunit, also known as PRKAA2) and phosphorylated (p-)mTOR (Ser2448), with GAPDH as a normalizing protein control (Fig. 5). Fig. 5A,B reveals a significant increase in the pAMPK protein level 24 h post DNA damage induction as compared to that in untreated and HC-hMSC controls. This increased level of pAMPK in ALS-hMSCs is blocked by the autophagy inhibitor 3MeA. Fig. 5C,D shows a concomitant and significant decrease in the p-mTOR protein levels in ALS-hMSCs 24 h post DNA damage induction, while a significant increase in p-mTOR levels is observed in HC-hMSCs. Again 3MeA blocks the 24 h NCS effect on p-mTOR inhibition by eliciting a significant increase in p-mTOR levels in ALS-hMSCs, which reached a similar level to that in HC-hMSCs. Overall the increase in pAMPK levels in ALS-hMSCs at 24 h post DNA damage induction is consistent with the concomitant decrease in p-mTOR levels observed in the disease cells. Interestingly, we found that the effect of DNA damage response in HC-hMSCs via pAMPK activation is negligible and p-mTOR inhibition is reversed, while the opposite is the case in ALS-hMSCs, emphasizing the abnormal regulatory mechanism controlling autophagy initiation pathway in the ALS cells.

## DISCUSSION

ALS covers a spectrum of neurodegenerative disorders, characterized by progressive degeneration of MNs (Silani et al., 2011). ALS has a multifactorial origin with high genetic background and clinical outcome diversity among patients; remarkably, in this study we managed to reveal common phenotypic differences in sALS patient cells in response to induced DNA damage. The four ALS biomarkers that were found previously by us in peripheral blood lymphocytes and hMSCs of sALS patients may indicate, at the gene expression level, that there are common disease-related pathways and specific genes affected in different non-neuronal tissues of ALS patients. Together with this, MN cell death in ALS is also suspected to be due to DNA damage caused by oxidative stress (Martin et al., 2006). Molecular evidence for such a response to DNA damage was found in the brain cortex, spinal cord, plasma and urine samples of ALS patients (Ferrante et al., 2002; Bogdanov et al., 2000; Kikuchi et al., 2002), and there is also evidence for excessive damage in somatic and mitochondrial DNA in the brain and spinal cord of transgenic mSOD1^G93A^ mice, even at the preclinical stage of 60 days (Sau et al., 2007; Kisby et al., 1997; Warita et al., 2001; Aguirre et al., 2005). Tandan and colleagues have studied the survival and DNA repair capacity in skin fibroblasts from sALS patients after treatment with several DNA-damaging agents, such as ultraviolet light, X-rays and mitomycin C, producing no significant difference in mean survival between ALS and control cells. However, they found that in response to DNA alkylating agents (which cause intra-strand linking) both cell survival and DNA repair were significantly reduced in ALS-derived fibroblasts (Tandan et al., 1987). In line with these findings, DNA damage induced by NCS in ALS-hMSCs caused a differential decrease in cell viability as compared to that seen in HC cells. Surprisingly, we found that this the induced cell death pathway in the ALS cells is only mediated by autophagy and is probably triggered by pAMPK activation that is blocked by the upstream 3MeA autophagy inhibitor. Generally, in other cell (non-ALS) models, cell death is induced as a result of NCS-mediated DNA damage in a dose-dependent manner (Shiloh et al., 1982) mostly by triggering apoptosis through the p53 and caspase cell death pathway (Benosman et al., 2007). Interestingly, in our NCS-mediated DNA damage experiments, we did not see any difference in p53 expression levels in the ALS cells that could be correlated with the gradual increase in cell death observed during 24 h after NCS treatment. Although the apoptotic cell death pathway has been studied as the major cellular response to DNA damage, recent reports on ionizing radiation (IR) and NCS-induced DNA damage on cancer cells suggest that autophagy also plays an important role in determining cell fate (Rodriguez-Rocha et al., 2011; Guerra, 2012), by acting either as a death mediator through autosis (Liu and Levine, 2015) or as a cytoprotective mechanism exerting futile attempts for cell rescue in face of cell injury and cell death. We found in our NCS-mediated DNA damage ALS-hMSCs model an overall increase in the autophagy marker LC3 by WB analysis at 24 h post NCS induction, representing more than 30% of the cell population, as judged by cell-based HCA of the LC3 integrated intensity levels. An increase of LC3-II over LC3-I in the ALS-hMSCs suggests that either autophagic induction or its late suppression, both of which lead to an accumulation of the lipidated form LC3-II, was induced, indicating an increase in intracellular autophagic activity (Mizushima et al., 2010). The differences in the expression of the autophagy markers induced by NCS-mediated DNA damage treatment in our experiments demonstrate that autophagy is abnormally regulated only in ALS cells. Interestingly, 3MeA can inhibit both class I PI3K activity (which inhibits autophagy) as well as class III PI3K activity (which is required for autophagy) (Knight et al., 2006). In our experiments, 3MeA decreased pAMPK activation as a result of NCS-induced DNA damage in ALS-hMSCs. pAMPK can activate class III PI3Ks, which eventually induce autophagy (Ouchi et al., 2004), but also inhibit, in parallel mTOR, therefore promoting autophagy in a more direct way (Rubinsztein et al., 2012). This strongly suggests that these mentioned pAMPK/mTOR pathways are mediating the autophagy response in ALS-hMSCs, and importantly that the ALS phenotype also causes abnormal regulation of autophagy induction, rather than acting on other controls along the autophagic pathways (i.e. autophagosome elongation, amphisome formation, autophagosome-lysosome fusion and lysosomal degradation; Klionsky et al., 2016). Moreover, studies conducted in the ALS mouse model mSOD1^G93A^ showed high pAMPK levels as a result of increased pATM in the spinal cord (Lim et al., 2012; Perera and Turner, 2016). The autophagy-mediated pathway in ALS-hMSCs might be induced as a result of induction of the DNA damage response through either pATM activation or through mitochondrial dysfunction via regulation of the mitochondrial levels of ATP and AMP (which would increase the AMP:ATP ratio and thus activate AMPK). ATM signaling to AMPK serves as a central regulator of protein synthesis and cell growth that can be activated by ATM in the cytoplasm to integrate reactive oxygen species (ROS)-mediated DNA damage response pathways with adequate metabolic signaling, protein synthesis and cell survival. Such a ROS-induced repression of mTOR triggering autophagy was described in a melanoma cell line, whereby ATM activates AMPK via the AMPK kinase and ATM effector LKB1 (also known as STK11) (Alexander and Walker, 2011). Supporting evidence for mitochondrial dysfunction in the ALS cells is based on a study that shows cell stress induced mitochondrial dysfunction in MNs from mSOD1^G93A^ ALS mice, as measured by impairment in the ATP:AMP ratio, which in turn increases pAMPK levels (Ferri et al., 2006; Israelson et al., 2010). Taken together, this evidence may support our findings in ALS-hMSCs where pAMPK mediates the autophagy response to NCS-induced DNA damage, possibly reflecting a common mechanism of disease between MN and hMSCs in ALS.

An interesting aspect of our results is the possibility that the significant decrease in p-p53Ser37 at 12 h and then the increase at 24 h post DNA damage induction in ALS-hMSCs might be related to the concomitant increase in pAMPK and autophagy observed in these experiments. It is known that a decrease in p-p53 levels as a result of cell stress can be associated with AMPK activation and mTOR repression (Lavin, 2008). A decrease in p-p53 levels enhance ATP consumption because the cells under stress are trying to compensate for energy consumption by activating the energy sensor AMPK signaling, which in turn inhibits mTOR, stimulating autophagy (Viana et al., 2008). The direct communication between the p53, pAMPK and mTOR pathways might play an important role in ensuring the right metabolism for normal cell growth and proliferation (Jing et al., 2011; Feng et al., 2007), supporting the view that deregulation of this axis might lead to a disease cell mechanism that eventually causes cell death.

Our innovative approach using patient-derived ALS-hMSCs has revealed important intrinsic phenotypic differences in the ALS cell response to DNA damage. These differences may shed some light on the cellular mechanism of ALS disease. Further studies on autophagy-mediated cell death and the DNA damage response in ALS need to be performed in relevant tissues and cells (brain, spinal cord and muscles) of the diverse ALS mouse models to determine their pathophysiological relevance. This kind of approach from patient cells to animal models was proven to be useful for the discovery of ALS biomarkers that were first identified by us in hMSCs of sALS patients (Lilo et al., 2013). In addition, this autophagy-mediated cell death pathway in ALS-hMSCs may be a platform for personalized high-throughput drug screening to find beneficial and more accurate therapies for patients suffering from this incurable and devastating disease.

## MATERIALS AND METHODS

### Bone marrow samples: isolation and culture

Bone marrow samples were obtained under general light anesthesia by aspiration from the iliac crest of seven healthy donors (age range: 18–54 years) and ten ALS patients (age range: 41–68 years). Written consent of all volunteers was collected before sample donation according to the guidelines of the ethics committee of the Laniado Hospital and The Hadassah Medical Centre supervised by the Israeli Health Ministry Ethics Committee conforming with The Code of Ethics of the World Medical Association (Declaration of Helsinki), printed in the British Medical Journal (18 July 1964). The hMSC samples used in this study were the same as those originally isolated from bone marrow samples, and were phenotypically characterized by fluorescence-activated cell sorting (FACS) and by assessing the multipotency differentiation potential to bone, fat and cartilage cells, as previously described by us (Nachmany et al., 2012). Bone marrow hMSCs samples from up to a total of seven healthy controls and six sporadic ALS patients at passages 3 to 9 were used for the different experiments in this study. Owing to the limited number of samples available for the experiments in this work and the limited number of passages that these cells can be used for testing, some experiments were performed with less cell samples than other experiments as indicated for each figure in the results section.

### NCS-mediated DNA damage induction and cell viability HCA assay

The hMSCs were cultured at 37°C in a 5% CO_2_ incubator until reaching 80% confluence in culture medium consisting of DMEM/F12 (Biological Industries, IL) with 10% fetal bovine serum (FBS; Hyclone, Gibco, UK), 1% gentamycin (Biological Industries) and 1% penicillin-streptomycin-nystatin (PSN) (Biological Industries); then the cells were briefly trypsinized (0.25% Trypsin-EDTA solution B; Biological Industries), centrifuged at 132 ***g*** for 5 min and re-suspended for cell counting using an hemocytometer. 1500 cells were plated per well in microscopy-grade 96-well plates (Grenier Bio-One, GER) and allowed to grow for 24 h in culture medium. Culture medium was removed and replaced with serum-free medium for an additional 3 days before treatment. Neocarzinostatin (NCS; Sigma-Aldrich) solution at 50, 150, 300 and 500 ng/ml final concentration was added to the cells for the first 30 min of incubation and then replaced by new serum-free medium and cultured for a period of 24 h at 37°C in a 5% CO_2_ incubator. Alternatively, experiments were performed with 50 ng/ml NCS on hMSCs and evaluated at 1, 2, 4, 12 and 24 h. 10 mM 3-methyladenine (3-MeA) or 20 μM Q-VD-OPH (both Sigma-Aldrich) was added for 24 h or 30 min, respectively, prior to NCS treatment in the relevant wells. For the cell viability assay, a mix of cellular fluorescent dyes in PBS was prepared, including Hoechst 33342 at 1:10,000, propidium iodide (PI) at 1:2000 (Sigma-Aldrich) and Calcein-AM at 1:5000 (Molecular Probes, MA), and 5 μl of this mix was added to each well for 30 min at 37°C in a 5% CO_2_ incubator before being transferred to an InCell2000 (GE Healthcare, UK) machine for image acquisition at 20× magnification. Images of the labeled cells were sequentially analyzed and segmented automatically for each fluorescence channel wavelength by using the InCell2000 developer software (GE Healthcare, UK), producing an output based on comparative fluorescence intensity. The percentage of viable cells was calculated by subtracting the number of PI-positive cells from the total number of nuclei counted in each well.

### Immunofluorescence analysis

After treatments (see above), the cells were washed with PBS and fixed with cold 4% paraformaldehyde (Electron Microscopy Sciences, PA) for 20 min. After rinsing three times with PBS (Gibco), cells were permeabilized using 0.1% Triton X-100 (Sigma-Aldrich) in PBS for 5 min at room temperature. Cells were incubated for 1 h in blocking solution consisting 5% FBS in PBS. Primary antibodies that were applied for 1 h at room temperature to the cells were diluted in blocking solution as follows: 1:200 rabbit anti-human γ-H2AX (Abcam, ab2893), 1:100 rabbit anti-human caspase 3 (cleaved) (Cell Signaling, 9661), 1:20 rabbit anti-human p-p53Ser37 (Cell Signaling, 9289), 1:100 rabbit anti-human pDNA-PK (Abcam, ab18192), 1:200 mouse anti-human pATM (Millipore, 05-740) and 1:50 rabbit anti-human LC3 (Sigma-Aldrich, L8918). Secondary antibodies used were 1:400 donkey anti-rabbit or mouse IgG conjugated to Alexa Fluor^®^ 488 or 1:400 goat anti-rabbit or mouse IgG conjugated to Alexa Fluor^®^ 594 (Life Technologies), and were applied after three PBS washes for a 1 h incubation in the dark at room temperature together with a mix of 1:10,000 Hoechst 33342 (Sigma-Aldrich) and 1:400 phalloidin conjugated to Alexa Fluor^®^ 594 (Life Technologies) to label all nuclei and F-actin. The cells were then rinsed with PBS, and visualization of immunofluorescence-labeled preparations was performed by using an InCell2000 machine with a 20× magnification objective. Images of the labelled cells were sequentially analyzed and segmented automatically to quantify the integrated intensity of the different immunofluorescent-labeled protein signals within the cells from the multiple images by using InCell2000 developer software (GE Healthcare, UK).

### Western blot analysis

Cells were grown in six-well plates (Corning) at a seeding density of 100,000 cells/well. The cells were cultured and treated with NCS alone or NCS in combination with 3-MeA or Q-VD-OPH for 24 h in a similar way to that described for the experiments above. For western blot preparations, the cells were washed twice with cold PBS for the removal of serum residues and dead cells. Cell lysates were obtained by using RIPA buffer (Sigma-Aldrich) in the presence of cOmplete protease inhibitor (Roche-Diagnostics, Basel, SW). 100 μl of RIPA was applied to 10^5^ cells for 5 min on ice. Protein content in samples was calculated by using a BCA protein assay kit (Pierce) according to manufacturer's instructions. 24 μg of denatured protein samples were loaded on 16% polyacrylamide (Bio-Rad) gels in Tris-HCl and SDS buffer consisting of 1 M Tris-HCl, 10% SDS, 10% ammonium persulfate and 1% tetramethylethylenediamine (TEMED; Sigma-Aldrich) and were separated by electrophoresis with Tris/glycine/SDS buffer (Bio-Rad). Protein transfer onto nitrocellulose membrane was performed in a blotting chamber (Bio-Rad) that was filled with premixed transfer buffer (Bio-Rad) for 1 h at 100 V in cold achieved by using an ice block. Membranes were then incubated for 1 h in WB blocking solution containing 5% FBS in PBS and 0.1% Tween 20 (Sigma-Aldrich). Primary antibodies were diluted in WB blocking solution and then added for overnight incubation at 4°C at the following dilutions: 1:1000 rabbit anti-human LC3 (Sigma-Aldrich, L8918), 1:1000 rabbit anti-human mTOR (Cell signaling, 5536), 1:1000 rabbit anti-human p-AMPKα2 (Cell Signaling, 4188), 1:5000 mouse anti-human β-tubulin (Sigma-Aldrich, T8328) and 1:5000 mouse anti-human GAPDH (Cell Signaling, 5174). The membranes were probed with the secondary antibodies [1:10,000 horseradish peroxidase (HRP)-conjugated donkey anti-rabbit IgG (Abcam, UK) or 1:20,000 HRP-conjugated donkey anti-mouse IgG (Abcam, UK)] diluted in WB blocking solution for 1 h at room temperature, followed by three PBS washes. To detect the specific protein bands, ECL Super signal kit (Pierce Biotechnology) was used and luminescence signal detection imaging was performed with an Amersham Imager 600 (GE Healthcare). Quantification of band intensities was performed by densitometry analysis using Image J.

### Statistical analysis

*P*-values were calculated with a Student's *t*-test with equal distribution as determined by using the SPSS 20.0 for Windows statistical software package (IBM, USA). *P*<0.05 was considered statistically significant.
